# Book Reviews

**Published:** 1893-07

**Authors:** 


### Book Reviews.

HUMAN ANATOMY. A complete systematic treatise by various authors, including a special section on Surgical and Topographical Anatomy. Edited by HENRY MORRIS, M. A. and M. B., London, Surgeon to and Lecturer on Surgery, formerly Lecturer on Anatomy at the Middlesex Hospital; late Examiner in Anatomy in the University of Durham, and for the Royal College of Physicians on the Conjoint Board. Illustrated by 791 wood-cuts, 214 of which are printed in colors from drawings made expressly for this work by special artists. Philadelphia: P. Blakiston, Son & Co., 1012 Walnut street. 1893.

The first paragraph of the author's preface to this commendable work reads as follows: "This treatise on Human Anatomy is designed for the use of students, and aims at being a complete and systematic description of every part and organ of the body, so far as it is studied in the dissecting room." Even a hasty perusal of the contents of the volume convinces the reader at once that the author's prefatory statement is a correct one, for it is, indeed, "a complete and systematic description" of the gross anatomy of "every part and organ of the body," well worthy the careful and studious attention of practitioners as well as students proper.

The accuracy of description and thoroughness of detail in the work before us is no doubt due, in a large measure, to the fact that the editor, in preparing the book, has availed himself of the valuable services of many eminent authors who are known to have devoted special attention to the subjects allotted them. In looking over the arrangement of authors and subjects, we find many names which afford us an insight into the character of the work. The section on Osteology is attributed to J. Bland Sutton, F. R. C. S.; that on Muscles, to J. H. Davies-Colley, F. R. C. S.; that on Blood Vessels and Lymphatics, to Wm. J. Walsham, F. R. C. S.; that on the Nervous System, to H. St. John Brooks, M. D.; that on the Eye, to R. Marcus Dunn, F. R. C. S.; that on the Tongue, Nose, Ear, Heart, Throat and Voice, to Arthur Hensman, F. R. C. S.; that on Organs of Digestion, to Frederick Treves, F. R. C. S.; that on the Urinary and Generative Organs, to Wm. Anderson, F. R. C. S., and that on Surgical and Topographical Anatomy, to W. H. A. Jacobsen, F. R. C. S.

A glance at these names of ten of the foremost anatomists and surgeons of the English-speaking world impresses one with the conviction that Prof. Morris knew his men, and that their combined efforts have given us a treatise on anatomy which, for a long time to come, we believe, is destined to fill an important place as a students' text-book and work of reference.

The value of the book is greatly enhanced by the numerous illustrations which adorn its pages, over 200 of which are printed in colors from drawings made expressly for this work by special artists, who have spared no pains to make each cut clear and representative of the normal structures and relations. A few of the illustrations might be made subjects of criticism, but, on the whole, they are very accurate and add greatly to the utility of the work.

A feature of the book which will undoubtedly facilitate the work of students, is found in the illustrations of the bones, where the origin of the muscles are indicated by red lines, the insertions by blue lines, and the attachment of ligaments by dotted black lines.

So for as size, paper, and type are concerned, the book is all that could be desired, and taken all in all, the production is one which reflects much credit on both its authors and publishers.

E. C.

---

LECTURES ON MENTAL DISEASES, designed especially for Medical Students and General Practitioners. By HENRY PUTNAM STEARNS, A. M., M. D., Physician Superintendent of the Hartford Retreat, Lecturer on Mental Diseases in Yale University; Member of the American Medico-Psychological Association; Member of the New England Psychological Society; Honorary Member of the British Psychological Association; Honorary Member of the Boston Medico-Psychological Society; Member of the American Medical Association, etc., etc. With illustrations. Small octavo; pp. xviii.—636. Price, $3.00. Philadelphia: P. Blakiston, Son & Co., 1012 Walnut street. 1893.

This is an excellent work prepared as a basis for instruction in mental diseases to the medical students of Yale University. Its author, Dr. Stearns, Superintendent of the Hartford Retreat, is an authority in this branch of medicine, and one of the ablest members of the American Medico-Psychological Association.

The author has divided his matter very systematically into twenty-nine sections, and ending with an appendix on extracts from the laws of the different states and territories of the United States, which relate to the general care of the insane. The first section is devoted to a review of the anatomy of the brain and spinal cord, which, though not intended to be exhaustive, is, nevertheless, concise and comprehensive. In the succeeding chapters are considered the symptoms of insanity with themes as to their production. The classification of mental diseases has always been a bone of contention, and so far every writer has his own classification. The one followed by Dr. Stearns is very practical, and indeed excellent. He classifies the different forms into—

(*a*) Symptomatological, including (1) melancholia; (2) mania; (3) primary delusional insanity; (4) folie circulaire; and (5) dementia.

(*b*) Etiological, (1) epochal (physiological), including insanity of puberty, climacteric insanity, and senile insanity; (2) sympathetic (sexual), including puerperal, masturbatic and ovarian insanity; (3) toxic, including alcoholic and syphilitic insanity; (4) neuropathic, including epileptic and hysterical insanity; (5) pathological, including general paralysis, insanity from coarse brain disease, and acute delirium; (6) other less frequent genera and species, as phthisical insanity, rheumatic insanity, and post-febrile insanity.

The author has no time to lose on the term paronoia. He says concerning it: "It certainly has relations neither with a symptomatological, pathological, plysiological, or etiological basis of nomenclature, nor has it even the merit of a neutral character."

The writer then describes fully the various forms as enumerated in the classification, reciting cases when necessary, and pays special attention to the treatment, which, by the way, is unusually sound and exhaustive. The index on the legal care of the insane is an excellent idea and adds much to the value of the book.

The typographical work is hardly what one would expect such a book to be clothed in. It is certainly much too inferior for the high character of its printed matter. W. C. K.

---

Sexual Neurasthenia (Nervous Exhaustion). Its Hygiene, Causes, Symptoms, and Treatment, with a chapter on Diet for the Nervous. By George M. Beard, A. M., M. D., formerly Lecturer on Nervous Diseases in the University of the City of New York; Fellow of the New York Academy of Medicine; Author of Our Home Physicians, Hay Fever; one of the authors of Medical and Surgical Electricity, etc. (Posthumous manuscript.) Edited by A. D. Rockwell, A. M., M. D., Fellow of the New York Academy of Medicine, and Electro-Therapeutist to the New York State Woman's Hospital; one of the authors of Medical and Surgical Electricity, etc. Third edition. Pp., 282. Price, $2.75. New York: E. B. Treat, 5 Cooper Union. 1891.

Nervous Exhaustion (Neurasthenia.) Its Hygiene, Causes, Symptoms, and Treatment. By George M. Beard, A. M., M. D., formerly Lecturer on Nervous Diseases in the University of the City of New York; Fellow of the New York Academy of Medicine, etc. Second edition, revised and enlarged by A. D. Rockwell, A. M., M. D., Professor of Electro-Therapeutics in the New York Post-Graduate Medical School and Hospital; Fellow of the New York Academy of Medicine, etc. Pp. 254. Price, $2.75. New York: E. B. Treat, 5 Cooper Union. London: H. K. Lewis, 136 Grower street. 1892.

These two volumes on General Neurasthenia and Sexual Neurasthenia are, perhaps, the most important of the late Dr. Beard's productions.

These volumes have become authoritative throughout the scientific world, as evidenced by their translations, and perusal by continental and English physicians. It is fortunate that Dr. Rockwell, under whose direction these volumes are now edited, enjoyed the full confidence of his teacher, thus enabling him to make such necessary additions without injuring the original fabrics.

These volumes need no special review ; they have their place in modern scientific literature.

The publishers have succeeded admirably in making these books durable as well as handsome. W. C. K.

---

SYSTEM OF DISEASES OF THE EAR, NOSE, AND THROAT. Edited by CHARLES H. BURNETT, A. M., M. D., Emeritus Professor of Otology in the Philadelphia Polyclinic ; Clinical Professor of Otology in the Woman's Medical College of Pennsylvania ; Aural Surgeon to the Presbyterian Hospital, etc. Philadelphia, Pa.: Vol. I. J. B. Lippincott Company.

The reader of the above volume will be struck with its excellence. Its contents are complete, concise, and abreast of the times ; it is such a book as is needed by the student and practitioner. The authors of the various chapters are men peculiarly adapted to write on the subjects which have been assigned them. The text is well illustrated by the citation of many interesting and instructive cases.

The first part of the volume, 560 pages, is devoted to the diseases of the ear, and of this nearly a hundred pages are devoted to the anatomy and physiology of that organ, and yet the reader must feel that not too much has been said. This part of the text is well illustrated by well selected plates from the recent works of Politzer, Gruber and others.

The author of the chapter on Otitis Externa has devoted considerable space to otitis externa parasitica, which is very important in view of the intractable nature of the condition. Sir William Dalby strongly criticizes in the article on Foreign Bodies, the careless way in which practitioners, not in the habit of examining the ear, undertake the removal of foreign bodies from the external auditory canal, and states emphatically and truly that the operator, and not the foreign body, is often the cause of any harm to the ear which may result from its presence.

The presence of adenoid vegetations in the naso-pharynx is dwelt on at some length as a cause of aural troubles.

The author of the chapter on Otitismedia sums it up in these words :

> These frequent recurring acute and subacute attacks during childhood lead often to adhesions and permanent retraction of the drumhead, and in some cases the internal ear is secondarily involved. Acute catarrhal otitis media in children will be found to be due in a great number of cases, especially in those who are poorly nourished or debilitated, to the presence of adenoids in the post-nasal cavity.

Dr. Sexton's radical operation, *i. e.*, excision of the membrane and ossicles, is strongly advised for both chronic catarrhal and suppurative diseases of the middle ear. The results in both classes of cases are fully illustrated by the histories of numerous cases.

Dr. Burnett claims that no case of acute purulent inflammation will become chronic, *no matter what its cause*, if properly treated at the outset.

The second part of the volume treats of diseases of the nose and naso-pharynx.

The chapter on Anatomy is extensively illustrated by plates from Hirschfeld and Leuschka. The last chapter of the volume is devoted to diseases of the accessory sinuses of the nose, a department of rhinology which has received a great deal of attention in the last four years. Dr. Bryan has written a most excellent article on the subject, the most complete that we have seen in any text-book.

W. S. R.

---

HAND-BOOK OF INSANITY FOR PRACTITIONERS AND STUDENTS. By DR. THEODORE KIRCHHOFF, Physician to the Schleswig Insane Asylum, and Privatdocent at the University of Kiel. Illustrated with eleven plates. Octavo volume, pp. 362. New York: William Wood & Co. 1893.

This work is divided into two parts, the first, a general part, deals in Chapter I. with the anatomical basis and the location of mental disturbances.

Chapter II. considers the causes of insanity as civilization, race, and geographical position, sex, social position, age, and heredity. Of this latter cause he says: "It is now regarded as certain that the influence of the father in heredity is more important than that of the mother." What he says of suicide in this connection is interesting; the most striking phenomenon in this respect is the inheritance of the tendency to suicide. Cases are reported in which one member of a family after another committed suicide, although living in remote parts of the world. Suicide on an hereditary basis is found even in children at the age of five years. The other chapters of this part deal with the signs and course of mental disorders and their diagnosis.

Of post-mortem findings he says: "These possess very little value as an aid in the confirmation of diagnosis in insanity."

The chapter on Treatment is good, so far as it goes, but it is lacking in all reference to the modern means of mental exercise and diversion by means of regular employment and amusements.

Part two considers the special forms of insanity, describing them very fully and well. Clearness of style is marked throughout the book, and assures for it great popularity. J. W. P.

---

INTERNATIONAL CLINICS. A Quarterly of Clinical Lectures on Medicine, Neurology, Pediatrics, Surgery, Genito-Urinary Surgery, Gynecology, Ophthalmology, Laryngology, Otology, and Dermatology. By professors and lecturers in the leading medical colleges of the United States, Great Britain and Canada. Edited by JOHN M. KEATING, M. D., LL. D., Colorado Springs, Col., Fellow of the College of Physicians, Philadelphia ; formerly Consulting Physician for Diseases of Women to St. Agnes' Hospital, Gynecologist to St. Joseph's Hospital, Visiting Obstetrician to the Philadelphia Hospital, and Lecturer on Diseases of Women and Children, Philadelphia ; Editor Cyclopedia of the Diseases of Children. Judson Daland, M. D., Philadelphia, Instructor in Clinical Medicine and Lecturer on Physical Diagnosis and Symptomatology in the University of Pennsylvania ; Assistant Visiting Physician to the University Hospital ; one of the Examiners of the Insane to the Philadelphia Hospital ; Visiting Physician to St. Clement's Hospital. Philadelphia. J. Mitchell Bruce, M. D., F. R. C. O., London, England. Physician and Lecturer on Therapeutics at the Charing Cross Hospital. David W. Findlay, M. D., F. R. C. P., Aberdeen, Scotland, Professor of Practice of Medicine in the University of Aberdeen ; Physician to, and Lecturer on Clinical Medicine in the Aberdeen Royal Infirmary ; Consulting Physician to the Royal Hospital for Diseases of the Chest, London. Volume IV. Second series. 1893. Philadelphia : J. B. Lippincott Co.

The present volume continues this interesting group of lectures much on the same lines as heretofore. The book opens with a memorial of Dr. Henry I. Bowditch, written by Frederick I. Knight. This author has done his work well. Dr. Bowditch's amiable character was of household knowledge wherever his name and fame extended, and it is delicately recorded by Dr. Knight.

One of the interesting lectures in this series is on Angina Pectoris, by Dr. Thomas J. Mays. This is a subject which demands more attention than it usually receives, and we are pleased to find that this author has thrown considerable light upon it.

In this volume Buffalo is represented by Dr. Charles Cary, who discourses upon Acute Croupous Pneumonia and Rheumatism, with Cardiac Sequelae ; Dr. Matthew D. Mann, who lectures upon the After-Treatment of Laparatomies ; Dr. Roswell Park, who treats of Fractures of the Cervical Vertebræ ; Dr. James W. Putnam, who discourses upon Neuritis, and Dr. Charles G. Stockton, who lectures upon Gastric Ulcer. These subjects are well presented by their several authors.

This volume is illustrated with a few photographic reproductions and a number of wood-cuts, but we think it would be well for authors to pay more attention to illustrating their clinics. Where they are preserved in such substantial form, the incentive to good illustrations should be sufficiently strong to accomplish the fact.

---

SYPHILIS AND THE NERVOUS SYSTEM, being a revised reprint of the Lettsomian Lectures for 1890, delivered before the Medical Society of London. By W. R. GOWERS, M. D., F. R. C. P., F. R. S., Consulting Physician to University College Hospital, Physician to the National Hospital for the Paralyzed and Epileptic, etc. Price, $1.00. Philadelphia: P. Blakiston, Son & Co., 1012 Walnut street. 1892.

These lectures have been reproduced in book-form, at the request of many of Dr. Gower's friends, and dwell upon a subject concerning which text-books have little to say.

The subject is divided into three parts: Chapter I., discussing the Ultimate Pathology of Syphilis; Chapter II., the Origin of Functional Nervous Disorders, attributed to Syphilis on Imperfect Evidence, and Chapter III., the Essential Principles Underlying the Prognosis of Syphilitic Disease of the Nervous System, and their Effect upon the Special Prognosis of the Chief Lesions. Upon the disputed relation between syphilis and tabes the author takes a strong stand in favor of such relation. He says: "Although the *method* which discloses syphilis may be open to question, I cannot but think that the observers who describe it in seventy-five or eighty per cent. *are not far from the real truth*." The degenerative ocular palsies are also connected with syphilis, and the author states that there is also strong evidence of a similar relation in simple atrophy of the optic nerve. As to general paresis, the writer is not quite so positive.

Chronic muscular atrophy, due to degeneration of the motor cells, is considered as an occasional consequence of syphilis. The other common degenerative diseases of the spinal cord are associated with syphilis only in a very small degree.

The little volume is well worth careful study by students of neurology and syphilology, and even the general practitioner will find in it much useful information not found elsewhere. W. C. K.

---

FISSURE OF THE ANUS AND FISTULA-IN-ANO. By LEWIS H. ADLER, JR., M. D., Instructor in Diseases of the Rectum in the Philadelphia Polyclinic and College for Graduates in Medicine. Detroit, Mich.: Geo. S. Davis. 1892.

This little monograph, published as one of the Physicians' Leisure Library series, is full of good, sound doctrine relative to the pathology, diagnosis, and treatment of the two very common and unpleasant maladies indicated in its title.

A study of the book reveals that it was written by one who has a practical knowledge of his subject. For this reason, as well as for others, its careful perusal will pay anyone who is called upon to manage the disorders of which it treats. It is presented to us in a neat and attractive attire, well printed on good paper, containing a number of cuts which add greatly to its value. E. C.

---

HISTOLOGY, PATHOLOGY, AND BACTERIOLOGY. A Manual for Students and Practitioners. By BENNETT S. BEACH, M. D., Lecturer on Histology, Pathology, and Bacteriology, New York Polyclinic. Series edited by BERN B. GALLAUDET, M. D., Demonstrator of Anatomy, College of Physicians and Surgeons, New York; Visiting Surgeon Bellevue Hospital, New York. The Students' Quiz Series, pp. 165. Price, $1.00. Philadelphia: Lea Brothers & Co.

This little volume is exceedingly useful in colleges requiring a two-year course only, but in the progressive institutions requiring a four-year course, it is very much out of place. Such volumes only prevent the students from purchasing the authorities on the special subject, and are hence not to be commended to the general student public. The book is divided into three parts: Histology, Pathology, and Bacteriology. Questions are arranged and the answers follow. It is all that the editors claim for it—to save time for the student and busy practitioner. The presswork is very neatly done. W. C. K.

---

THE INTERNATIONAL MEDICAL ANNUAL and Practitioner's Index for 1893. Edited by a corps of thirty-eight department editors—European and American—specialists in their several departments. P. W. Williams, M. D., Secretary of Staff. 626 octavo pages. Illustrated. $2.75. New York: E. B. Treat, publisher, 5 Cooper Union.

The Medical Annual for 1893 has made its appearance, and from nearly every standpoint excels its predecessors. The list of contributors comprises the ablest men of America, England, and Germany, thus making the Annual thoroughly representative of the views of all schools and countries.

As usual, the dictionary of new remedies claims first attention, followed by the dictionary of new treatment. The last chapter comprises the bulk of the work, the different diseases being arranged in alphabetical order. Among some of the novelties in new treatment may be mentioned æro-urethroscopy, or the electric light examination of the inflated urethra. The article is accompanied by three beautiful full-page plates, illustrative of the conditions present. The article on Laryngoscopy is also accompanied by three beautiful plates. The article on Sanitary Science is an important one, profusely illustrated, showing the latest improvements in sanitation. Progress in Pharmacy is ably treated by John Aulde, M. D. Then follow chapters on new instruments, new books, publishing houses, and a very complete index, making the Annual as accessible as could possibly be done.

The binding is similar to the other volumes of Treat's series, and forms a library worthy of a place in every physician's office.

